# An Electricity Price-Aware Open-Source Smart Socket for the Internet of Energy

**DOI:** 10.3390/s17030643

**Published:** 2017-03-21

**Authors:** Óscar Blanco-Novoa, Tiago M. Fernández-Caramés, Paula Fraga-Lamas, Luis Castedo

**Affiliations:** Department Electronics and Systems, Faculty of Computer Science, Universidade da Coruña, 15071 A Coruña, Spain; o.blanco@udc.es (Ó.B.-N.); tiago.fernandez@udc.es (T.M.F.-C.); luis.castedo@udc.es (L.C.)

**Keywords:** IoT, IoE, smart plug, energy monitoring, electricity-price awareness, home automation, power demand, HEMS

## Abstract

The Internet of Energy (IoE) represents a novel paradigm where electrical power systems work cooperatively with smart devices to increase the visibility of energy consumption and create safer, cleaner and sustainable energy systems. The implementation of IoE services involves the use of multiple components, like embedded systems, power electronics or sensors, which are an essential part of the infrastructure dedicated to the generation and distribution energy and the one required by the final consumer. This article focuses on the latter and presents a smart socket system that collects the information about energy price and makes use of sensors and actuators to optimize home energy consumption according to the user preferences. Specifically, this article provides three main novel contributions. First, what to our knowledge is the first hardware prototype that manages in a practical real-world scenario the price values obtained from a public electricity operator is presented. The second contribution is related to the definition of a novel wireless sensor network communications protocol based on Wi-Fi that allows for creating an easy-to-deploy smart plug system that self-organizes and auto-configures to collect the sensed data, minimizing user intervention. Third, it is provided a thorough description of the design of one of the few open-source smart plug systems, including its communications architecture, the protocols implemented, the main sensing and actuation components and the most relevant pieces of the software. Moreover, with the aim of illustrating the capabilities of the smart plug system, the results of different experiments performed are shown. Such experiments evaluate in real-world scenarios the system’s ease of use, its communications range and its performance when using HTTPS. Finally, the economic savings are estimated for different appliances, concluding that, in the practical situation proposed, the smart plug system allows certain energy-demanding appliances to save almost €70 per year.

## 1. Introduction

The world has many reasons to make the transition to a clean energy economy. Collectively, greenhouse gas emissions must be decreased in order to reduce significantly the risks posed by climate change. Some of the most dangerous consequences include intensifying droughts, storms, heat waves, the inundation of coastal economies brought on by rising sea levels, acidifying oceans, increasing wildfires and extreme weather events across the world. Reducing fossils dependence, tackling global warming, strengthening energy security and, thus, improving our health are today’s main concerns. Governments have set ambitious goals regarding the increase of renewable energy production, energy savings and greenhouse gas emission reduction in 2030 EU Energy Strategy [1] and the intended nationally-determined contributions to the Paris agreement [2]. There is no doubt that energy production systems need to be changed, since today they rely heavily on oil, natural gas and coal for generation. Nevertheless, rather than focusing only on new sources for energy supply, reducing and optimizing the demand should also be explored, as it would bring similar benefits.

The remarkably rapid growth of inexpensive and tiny computing devices, endowed with augmented sensing and communication capabilities, is paving the way for a growing presence and use of smart devices under the Internet of Things (IoT) paradigm: the number of IoT devices is expected to exceed 30 billion by 2020. Such devices are prone to security issues [3]; they draw data from large-scale industrial assets and other equipment and will increase global energy consumption. Moreover, the rise of Industry 4.0 and Cyber-Physical Systems (CPS) [4] represent a windfall for the smarter grid, which will operate in combination with the Internet of Energy (IoE). IoE [5] changes the concept of energy infrastructure, which is increasingly becoming dynamic, effective, intelligent, responsive and interconnected. Consequently, it reveals a promising future for eco-smart practices [6], more intelligent infrastructures (e.g., homes [7] or buildings) and industrial sectors, like defense [8,9], where electrical power systems would work cooperatively with smart devices to increase the visibility of energy consumption and create safer, cleaner and more sustainable energy systems. IoE systems require the use of multiple software and hardware components like embedded systems, power electronics, integrated circuits, sensors, processing units or storage technologies. Such components are an essential part of the infrastructure of companies dedicated to the generation and distribution energy and the one required by the final consumer.

In Spain, new regulations about energy cost became effective on 1 April 2014 [10]. Such regulations indicate that consumers with a contracted power of less than 10 kW can opt for the Volunteer Price for the Small Consumer (Precio Voluntario para el Pequeño Consumidor (PVPC) in Spanish) [11]. In this new pricing system, the price of the energy depends on the moment when it is consumed and charges are applied through smart meters. The installation of such in-home smart meters also allows consumers to know in real time the cost of the electricity they are consuming. In this way, theoretically, the supply should be more stable, the production costs would be reduced and the risk of fail or blackout due to power overloads should decrease.

This article presents a smart socket system that makes use of sensors, actuators and wireless transceivers to collect the information about pricing and the user preferences and to optimize home energy consumption. The system collects daily the data on pricing published by Red Eléctrica de España (Spanish Electrical Grid) and analyzes them to determine the time intervals with the lowest prices.

To this end, an autoconfigurable easy-to-deploy system has been developed. It is based on a series of intelligent power outlets connected to each other through a shared Wi-Fi network. Such power outlets measure the current of the devices connected to them by means of current intensity sensors. The collected data are sent to a central control device to generate statistics and to perform actions depending on the data. To carry out these actions, each socket embeds a relay that acts as an actuator and may allow or deny the passage of current to an appliance according to the commands sent by the central control device.

Thanks to the distributed allocation of smart sockets, energy consumption can be collected easily and monitored through a user-friendly interface to improve price-aware decision-making. Specifically, the advanced IoT-based structures presented in this article interact with the surrounding environment in terms of sensing and metering, processing this information to extract knowledge for optimizing energy efficiency at the demand side. Therefore, this article shows the benefits and opportunities that IoT technologies can provide for IoE.

It is important to emphasize the three main contributions provided by this article:

- It presents what to our knowledge is the first smart plug that manages in a real-world scenario the price values obtained from a public electricity operator to determine the time intervals with the lowest prices.
- One of the few open-source smart plug systems described in the literature is detailed.
- A novel wireless sensor network communications protocol based on Wi-Fi is presented that allows for creating an easy-to-deploy system that self-organizes and autoconfigures to minimize user intervention.

The remainder of this paper is organized as follows. Section 2 analyzes the latest academic and commercial smart power outlets. Section 3 details the design of the system proposed. Section 4 describes the communications architecture and the protocols implemented. Section 5 enumerates and gives details on the components of the system. Section 6 describes the most relevant elements of the software implemented. Section 7 details the experimental setup and the tests performed. Finally, Section 8 is devoted to the conclusions.

## 2. Related Work

### 2.1. Academic Developments

In the last few years, many researchers have studied different aspects of the monitoring and control of home energy. For example, a energy monitoring system using Bluetooth and GSM is introduced in [12]. Such a system consists of a central server that can be managed via SMS (Short Message Service) and that communicates through Bluetooth with a microcontroller, which is in charge of activating and deactivating electric power. A similar system is presented in [13], although it makes use of Power Line Communication (PLC). The authors take into account the existence of a solar accumulation system, whose non-critical loads are deactivated when the level of the accumulators is too low. The same authors present in [14] a similar system that controls remotely an Uninterruptible Power System (UPS). As in [13,14], PLC interfaces are embedded into the designs proposed in [15,16]. Another power outlet system based on PLC is presented in [17]: it can control the state of the outlets, and it is able to issue warnings about possible power overloads. In [18], the researchers replace the local controller with a cloud service in order to share resources and reduce costs, as well as to improve remote access for users.

In addition, the literature contains the work of researchers that have devised different smart plugs. For instance, a socket that uses an Ethernet module and an Arduino-Android platform is introduced in [19]. The researchers indicate that energy savings of 15% can be obtained thanks to their system. An Android-based application is also proposed by Horvat et al. [20], who present a Bluetooth Low Energy (BLE) solution for controlling appliances and monitoring power consumption. Another intelligent outlet that includes an Ethernet module is presented in [21]. Such an outlet is designed to work in a network where most of the energy comes from renewable sources.

Furthermore, other researchers [22] present a home controller system that makes use of a smart plug to monitor energy consumption efficiently using a serial to RS-485 converter to connect to the network. Subsequently, they provide an analysis of the energy consumption of a pilot house, based on a hypothetical scenario. The results present positive impacts on the energy consumption rate. Different methods are applied on emulated data to obtain gains between 10% and 22% in the energy consumption load.

Regarding the different communication technologies, several monitoring systems using a Wireless Sensor Network (WSN) based on ZigBee are described in the literature [23,24,25]. In the case of [23], the system proposed allows for remote switching off or on and for consulting its consumption in real time through a Graphical User Interface (GUI). Another similar system that uses ZigBee is [26], which also introduces the concept of reducing the consumption of devices in stand-by mode and uses various sensors to detect when an appliance is consuming more energy than it should in normal operation. Moreover, the authors of [27] also use ZigBee and include appliance detection via Radio Frequency IDentification (RFID), adding security features and device-specific monitoring capabilities.

Other authors propose the use of Bluetooth as a enabler of the smart management system. An example is [28], where a smart office is presented that can control the power state of a user’s PC and the switching off the lights through a location-aware approach based on BLE beacons, smart plugs and a mobile app. The experimental results obtained over a three-month period showed average energy savings of 31.9% for the PCs and 15.3% for the lights.

There are just a few developments in the literature that make use of Wi-Fi technology. For example, Thongkhao et al. [29] propose a plug that employs a low-cost Wi-Fi controller that contains a microcontroller and a Wi-Fi module. Besides, a bi-stable (latching) relay is employed to reach zero consumption in the relay coil when it is stable. The authors compared measurement accuracy with a reference meter and obtained an error of less than 0.5%.

The optimization of demand-based energy planning systems has been analyzed in the literature, as well. For instance, a communications protocol that considers scheduled and real-time devices is defined in [30]. In such a protocol, one of the devices assumes the role of the master and is in charge of coordinating the rest. Time is divided into slots, in which the devices negotiate the energy that they will be able to use in the next slot. A similar planning is described in [31], where devices are classified into switchable and non-switchable, and consumption planning is based on pricing in order to reduce the total cost.

Recent research studies have also been performed on the identification of the different home appliances. Ridi et al. [32] focused on analyzing classification algorithms, including K-Nearest Neighbor (KNN) and Gaussian Mixture Models (GMM), to recognize electric appliances automatically. The authors propose a system based on low-cost smart plugs that measure current periodically and that produce time series that characterize the consumption of an appliance. Thus, such electric signatures can be used to identify the type of appliance in use. Their best combination of features and classifiers shows a 93.6% accuracy. In 2015, the same authors [33] adopted machine learning approaches for the identification of appliances through their electric signatures. In addition, the researchers presented a database of 450 signatures that contained different brands and appliance models. Another machine-learning approach that allows for detecting configuration changes of smart plug installations is presented in [34].

Residential demand is also a thoroughly-studied topic, since it represents a significant portion of the total system load. Thus, residential Demand Response (DR) programs are important from the system operator’s perspective, and Home Energy Management System (HEMS) is an integral part of a smart grid that can potentially enable DR applications for residential customers. A HEMS is responsible for monitoring and managing the operation of in-home electrical appliances, providing load shifting and shedding according to a specified set of requirements. A possible solution for the management in already existing infrastructures is the use of smart plugs. For instance, an example of a smart socket with voltage modulation is presented in [35]. Such a device is able to support a fully-decentralized voltage service to control the residential electrical loads. Furthermore, Elma et al. [36] presented a smart plug designed to provide power reduction without turning the plugged device off, since their voltage is controlled automatically to avoid consumption peaks. The smart plug proposed communicates with an HEMS through a gateway that can measure and collect data about current, voltage or power. The communications between the plug and the HEMS interface are carried out using ZigBee. According to the researchers, their system reduces about 18% home peak demand for passive loads through voltage control. A different approach is taken in [37], where the authors make use of a data acquisition system to control the state of the outlets and to monitor current consumption.

The inclusion of HEMS in existing electrical appliances has also been studied. Tsunoda et al. [38] proposed a small-sized electrical power sensor that can be easily installed in home outlets. The sensor can measure the power of existing electrical appliances down to 1 W, and it is able to harvest energy from the power line. The power consumption of the sensor is almost 1/100th of that of conventional products.

Several studies about HEMS have been published recently. For instance, an energy consumption schedule for controllable appliances is described in [39]. Moreover, two different approaches for smart plug scheduling in an HEMS for DR programs with a time-of-use tariff are presented in [40]. One of the approaches makes use of centralized information received from several smart plugs that communicate with a home automation controller. The second approach is decentralized and runs locally on each smart plug. Another interesting work is presented in [41], where an intelligent DC power monitoring system is proposed. The system uses open-source software and guarantees that 10%–15% power savings are achieved with proper setting and scheduling. Similar systems based on ZigBee are described in [42,43]. Moreover, an integrated solution is proposed in [44], where the system enables small residential consumers to provide DR services for grid support considering both local energy resources and end-user’s convenience in a real-household environment.

When an HEMS gets smarter and adapts to the surrounding environment, it is called SHEMS (Smart HEMS). An SHEMS can make dynamic adjustments during the operation of home appliances to reduce energy cost, but as a result, it can affect the Quality of Experience (QoE) perceived by the user. This issue is tackled by some SHEMS, like the one described in [45], where the researchers propose a system that makes use of different appliance usage profiles to harness renewable energy resources and reduce energy cost by scheduling the tasks to be performed during off-peak hours. Another interesting SHEMS is presented in [46]. Such a system is controlled by an algorithm that optimizes the load scheduling process depending on price and energy consumption. As an example, the researchers model the thermodynamic process of a water heater considering explicitly user comfort as a constraint. Similarly, Jo et al. [47] focus on Heating Ventilation and Air Conditioning (HVAC) scheduling, incorporating in their model customer convenience whilst minimizing the overall energy cost of electricity and natural gas. Moreover, an interdisciplinary approach to a decoupled DR strategy and a learning-based HEMS is described in [48]. Finally, it is worth mentioning a human-centric SHEMS that integrates ubiquitous data sensing from the physical and cyber-spaces to infer patterns of power usage dynamically [49].

### 2.2. Commercial Systems

Today, there are numerous commercial smart power outlets on the market. Most of them were designed as plug-in adapters that act as an intermediary between the device and the power source. However, in terms of features, all of them are still far behind the academic systems mentioned earlier.

For instance, Belkin’s WeMo [50] uses Wi-Fi and allows users to switch on or off devices connected to it from a mobile application. Orvibo’s S20 [51] is very similar to Belkin’s device and allows for assigning timers to turn off and on power outlets. MyD-Link DSP-W215 from DLink [52] also makes use of a Wi-Fi network and adds a current sensor that lets users see the real-time consumption of a power outlet from a mobile application. MeterPlug [53] is aimed at measuring energy consumption, allowing for turning power outlets on and off manually and adding the functionality of stopping the current from flowing when the device is in stand-by mode. SafePlug [54] can switch on/off the power, monitor current consumption in real time and identify appliances to prevent fires and electrical shocks. Likewise, Edimax’s SP-1101W Smart Plug [55] uses Wi-Fi, and it also has an embedded power meter. Additionally, it promises savings with insight into how much you spend on each appliance and when it should be switched off (over-budget alerts). Power can be switched off automatically when a user-defined usage limit is reached.

Other devices worth mentioning are MyPlug 2 from Orange [56], which uses GSM (SMS); PlugWise [57] and SwannOne’s SWO-SMP1PA Smart Plug [58], which use ZigBee; MyModlet from Thinkeco [59] is a Wi-Fi enabled solution; and Ankuoo’s Neo Smart Plug [60], which makes use of 3G communications. Table 1 compares the most relevant features of the commercial devices previously mentioned. It can be observed that none of them makes use of real-time pricing to reduce energy cost.

Overall, it can be concluded that current commercial devices provide very similar and basic functionality (i.e., they can remotely turn on and off the outlets, monitor energy consumption and set schedulers and timers), but they are a step behind academic developments.

### 2.3. Analysis of the State-Of-The-Art

After analyzing all of the references mentioned previously, it is clear that most of the work carried out on smart plugs, especially on commercial sockets, has been focused on being able to control the switching on and off of devices remotely. More recently, other features have been added, like consumption monitoring [52,53], but only a few actually study the problem of implementing a planning system based on the price of energy to achieve cost savings and/or a reduction of the energy consumption [30,31]. However, note that such planning systems analyzed are limited to theoretical simulations: a real-world implementation of specific scheduling strategies has not been found in the literature as is described in this paper. Moreover, to our knowledge, no academic or commercial development has presented so far a hardware prototype that manages in a practical real-world scenario the price values obtained from a public electricity distributor.

Regarding the technologies used, most of the implementations use wired networks or point-to-point communications using wireless technologies. The latest developments use mesh sensor networks through ZigBee. Although ZigBee transceivers have been used extensively for creating WSNs in different fields [61,62,63], they are still relatively expensive, and they require the use of gateways to communicate through IP-based networks. It is worth mentioning that the ZigBee Alliance developed an open alternative called ZigBee IP [64], which includes IP connectivity through 6LoWPAN (IPv6 over Low-Power Wireless Personal-Area Networks) [65]. There are several manufacturers that are already selling ZigBee IP-compliant platforms, but it is not as widespread as the original ZigBee. Nonetheless, the price of Wi-Fi modules, like the one used by the solution proposed in this article, enables the creation of an extensible network, adding the possibility of connecting heterogeneous devices such as smartphones or tablets directly to the network.

Finally, no Wi-Fi smart plug that offers self-organizing mesh networking and the auto-configuration characteristics provided by the power outlet system presented in this paper has been found in the literature.

## 3. Design of the System

The system proposed in this paper allows for monitoring in real time the consumption of each connected device and storing the historical consumption values. Additionally, it makes it possible to remotely control the switching off and on of each of the power outlets and to plan the period of operation of each appliance in the most appropriate time slots to minimize the cost of energy consumption based on the daily price data.

**Figure 1:**
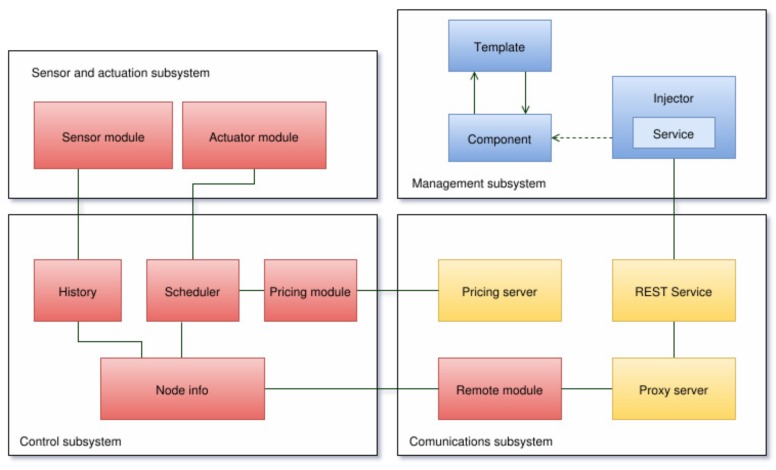
Subsystems diagram.

The system takes advantage of the fact that many appliances have some flexibility about when they should operate. For example, a user generally does not care when the washing machine works as long as the task is performed for a limited period of time and the laundry is ready before a time limit. Taking these parameters into account together with the hourly cost of energy, the system proposed can coordinate all of the devices to operate within the user’s preference ranges, selecting automatically the operation instants so that cost is minimized. The smart socket system is composed of the following subsystems, which are depicted in Figure 1:

- Sensor and actuation subsystem: This controls the sensors and actuators of the system. Such elements consist mainly of a current sensor and a relay. The subsystem is responsible for collecting current data and for activating the power outlet when it receives a request from the control subsystem.
- Communications subsystem: This consists of wireless transceivers that join an auto-configurable star topology.
- Management subsystem: This provides the user with the possibility of obtaining the current status of all modules, modifying their configuration and acting directly on them remotely through a web interface. In addition, this subsystem also includes a Representational State Transfer (REST) API that is responsible for transforming and redirecting the requests and responses between the clients and the corresponding device network through a proxy server.
- Control subsystem: This is in charge of controlling and managing the remaining subsystems, registering all existing nodes in the network, processing the data through the appropriate scheduling algorithms in each case and sending the necessary information to each of the modules. In addition, it acts as the gateway of the network to connect to the Internet, and therefore, it is the entry point to the network for the management subsystem.

### 3.1. Electricity Pricing in Spain

From April 2014 onwards, Volunteer Price for the Small Consumer (PVPC) is the electricity price-setting system that has been introduced by the Spanish Government in the regulated market. It is applied to the electricity bill of those consumers whose contracted power does not exceed 10 kW.

The PVPC system modifies the method for calculating the price of producing electricity. Consumers pay for their consumption, over a billing period, an hourly price obtained in the electricity intraday market. Thus, the computation of the new prices is performed by calculating the cost of energy production based on the day-ahead, along with other concepts established in the new methodology approved by the government as established in regulation RD 216/2014 of 28 March [10].

Red Eléctrica de España (REE) is the operator of the Spanish electricity grid and informs citizens by providing a tool to help them to manage their power consumption more efficiently. Everyday, around 8:15 p.m., REE publishes on its website the electricity pricing schedule that will be applied in each of the 24 h of the following day. Those consumers who have installed a smart meter, which is able to measure current by the hour, have these new prices applied to their bill in accordance with their consumption throughout the day. Therefore, the information regarding hourly pricing schedules allows consumers with a smart meter to adjust their bill if they adapt their electricity consumption to the time slots when electricity is cheaper. Note that in Spain by 2018, 100% of residential consumers will have a smart meter installed, so hourly prices will be applied to the 16.2 million users who are covered by PVPC (60% of the nearly 27 million electricity supply contracts that are currently signed in Spain).

### 3.2. PVPC Bill Calculation

The invoicing of the PVPC is composed by the sum of the following billing terms related to power, active energy and passive energy:

- The annual power billing FPU is calculated as the product of the power consumed (Pot expressed in kW) and the price of the power for the PVPC (TPU, expressed in euros/KW and year), according to the following equation:
  (1) $$\begin{array}{c} FPU=TPU\times Pot \end{array}$$
- The active energy billing term for the corresponding billing period, FEU, is the result of multiplying the energy consumed during the billing period in each tariff period and the price of the corresponding energy term according to the following formulas: In the case of using an smart meter, the formula corresponds to:
  (2) $$\begin{array}{c} FEU=\sum_{billing\;period}\left[\left(E_{p}\times TEU_{p}\right)+\sum_{h\in p}\left(Ep_{h}\times TCU_{h}\right)\right] \end{array}$$
  where:
  – $E_{p}$ is the energy consumed during the billing period *p* (in kWh).
  – $Ep_{h}$ is the energy consumed during the hour *h* of the billing period *p* (expressed in kWh).
  – $TEU_{p}$ represents the price of the energy term of the PVPC of the billing period *p* (in euros/kWh).
  – $TCU_{h}$ is the term of the hourly cost of energy of PVPC for each hour *h* (in euros/kWh).
  Without a smart meter, the formula corresponds to:
  (3) $$\begin{array}{c} FEU=\sum_{billing\;period}E_{p}\times \left[TEU_{p}+\frac{\sum_{h\in p}\left(TCU_{h}\times c_{h}\right)}{\sum_{h\in p}c_{h}}\right] \end{array}$$
  where $c_{h}$ is the hourly coefficient of the adjusted consumption profile. Every week, REE calculates and publishes the coefficients for the following week.
- The conditions established for the application of the reactive energy billing term, as well as the obligations related to it, are established in regulation RD 1164/2001 of 26 October 2001 and determine the access tariff for the electricity transmission and for the distribution networks.

### 3.3. Minimizing FEU

FEU (the active energy billing term) can be optimized if the consumer has a contract whose electricity consumption can be adapted to the time slots during the day when electricity is cheaper. For such a purpose, a software module was designed aimed at finding the optimum operating instant for a given time interval so that the active energy billing term is minimized.

To obtain such a time instant, the energy cost function *f(x)* has to be determined by obtaining the pricing data from Red Eléctrica de España (REE) [66], and then, Equation (4) has to be minimized. In such an equation, an integral is defined between *t* and *t + d*, where *t* is the first time instant when the electrical device monitored is switched on and *d* is its operating time. Note in addition that *t* and *t + d* must belong to the interval.

(4) $$\begin{array}{cc} & Total\;Energy\;Cost=\int_{t}^{t+d}f\left(x\right)dx\;\;\;\;\;\;\;t,t+d\in interval \end{array}$$

**Figure 2:**
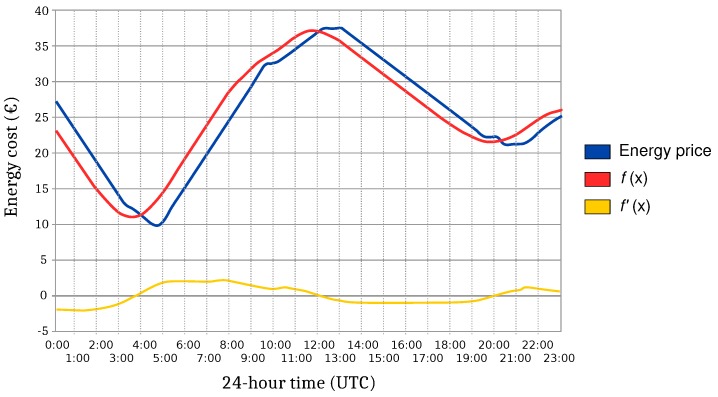
Example of the evolution of the energy prices during a specific time interval.

Assuming that the blue line in Figure 2 represents the cost of energy in a given time interval and considering a time interval of two hours, if the integral defined between *t* and *t + d* is calculated, with *d = 2* and *t* unknown, the function f(x) represented by the red line is obtained. This new function represents the energy cost of switching the device on for a period of 2 h at each of the time instants.

To find the optimal value, the value of *t* that minimizes f(x) has to be calculated. Note that the minimums and maximums of f(x) occur at points where the tangent to the function is horizontal. That is, the derivative $f^{\prime }\left(x\right)$ is zero at points *x* at which f(x) is maximized or minimized. Thus, the yellow line in Figure 2 represents $f^{\prime }\left(x\right)$, and it can be observed that it is equal to zero at three critical points around 3:30, 12:00 and 20:00. Analyzing f(x), it can be easily determined that the minimum is around 3:30.

The implementation presented in this paper follows the minimization steps previously described, but since f(x) is discrete, obtaining the minimum with a simple search is direct, and therefore, it is not necessary to calculate the first derivative.

## 4. Communications Protocol and Architecture

**Figure 3:**
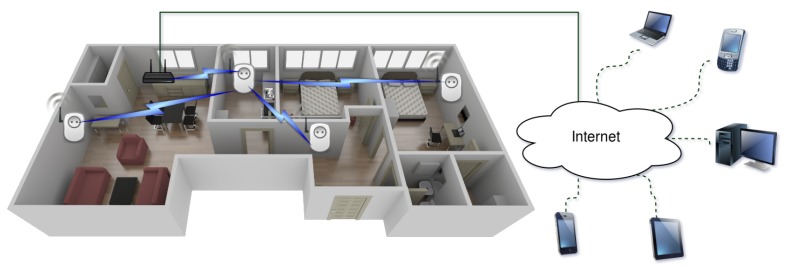
General overview of the system.

In Figure 3 is represented the communications architecture of the system proposed. The system distinguishes between two networks: an internal home network where smart plugs communicate with each other and an external network that allows remote users to access the internal network. Between both networks, there is a gateway that translates the external IP-based protocol to the internal ad hoc protocol. The next subsections describe how such protocols work and the inner workings of the communications architecture.

### 4.1. IoT Network

The IoT network is composed of nodes that connect to each other forming a star network topology, the central node being the one responsible for connecting to the gateway, which sends the data to the Internet.

Such a central node is designated as the master in the first phase of the negotiation for building the IoT network. All of the nodes that constitute the network have the ability to change their role and act as masters when required. However, the network self-manages to ensure that, at a specific time instant, only one of the nodes acts as the master. Thus, all nodes initially act as access points of a Wi-Fi network that allows for configuring each node individually and for discovering other nodes during the negotiation process of the network.

During the selection process of the master node, all nodes scan the network and compare the hardware identifiers of each device. Because such identifiers are assigned during manufacturing, it can be guaranteed that they are unique. When a node is powered up, it identifies all of the devices in the network with lower IDs and tries to communicate with them. Additionally, the device with the lowest ID changes its role to master and begins accepting connections from slaves. If after a certain number of attempts, it is not possible to establish a communication with the master node, the next device with the lowest ID is set automatically as the master until an agreement is reached. Once the negotiation has finished and the master is selected, all of the slaves connect to the Wi-Fi network offered by the master.

From this moment on, all communications between master and slaves will be carried out using HTTP. Thus, all nodes implement a lightweight HTTP server and an HTTP client. Although all modules were designed to support encryption using HTTPS, at this development stage of the prototype, all communications are performed in plain text to make debugging easier and to reduce development time. However, several tests were performed in order to assess the feasibility of using HTTPS (in Section 7.7), which would be essential in a final product.

#### 4.1.1. Keeping the Node List Updated

**Figure 4:**
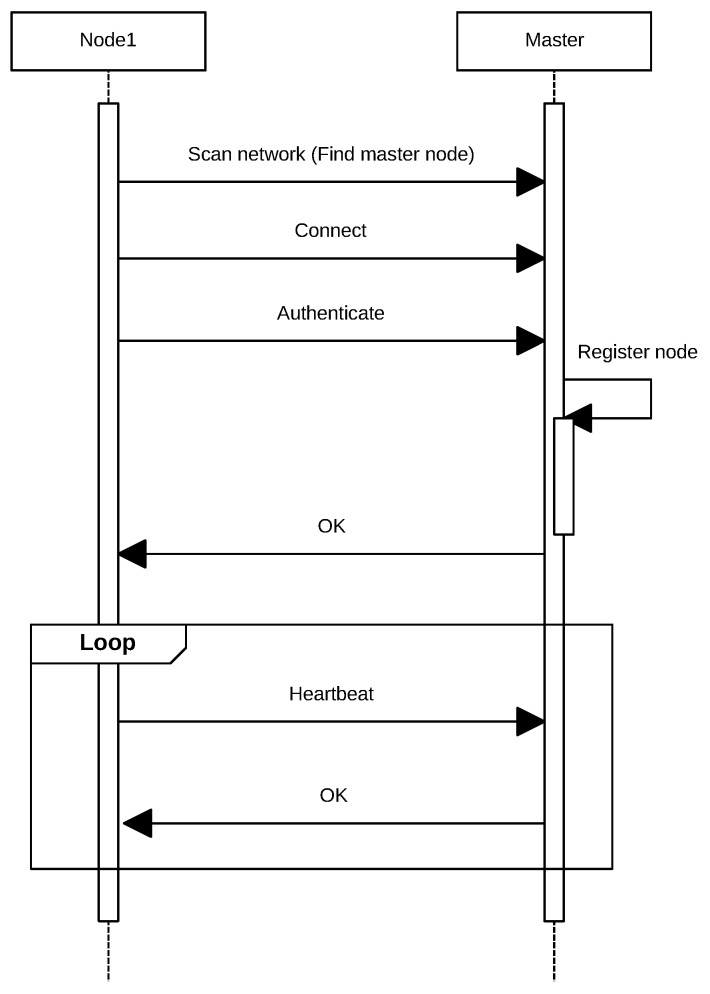
Sequence diagram for adding a new node.

All slave nodes report their status to the master by sending periodically a POST request (heartbeat) as illustrated in Figure 4. This process allows for keeping the list of available nodes updated.

With each heartbeat received, the master stores the ID of the node that sent the POST request and its IP. This information will be used to route incoming requests from the external network. If a node does not send heartbeats during a certain amount of time, the master considers that it is no longer available and deletes it from the list.

#### 4.1.2. Clock Synchronization

**Figure 5:**
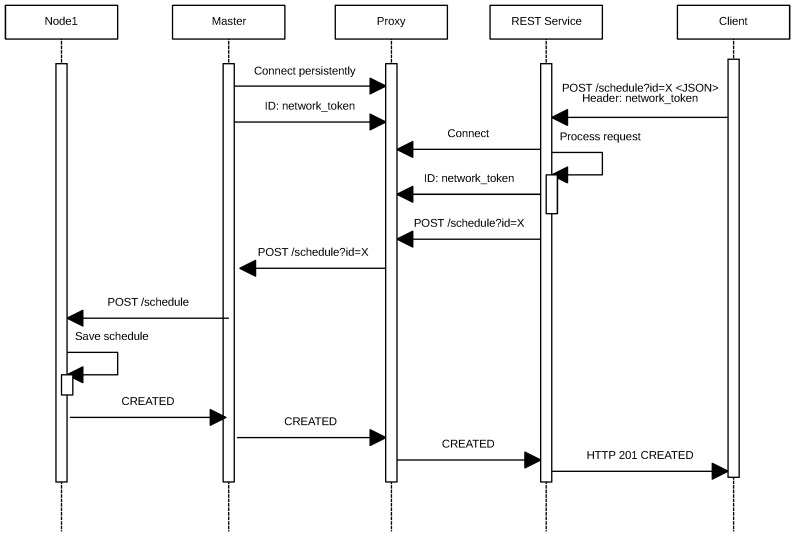
Sequence diagram for adding an operation interval.

The system proposed is based on creating an optimized energy consumption scheduling; therefore, it is essential that all nodes are synchronized and share the same time reference. To do this, the master node implements a Network Time Protocol (NTP) client that allows it to obtain the network time from an external server. The master node checks the time when it is initialized and at least once a day to make sure the network has the correct time. Regarding the slave nodes, they ask the master for the time periodically through HTTP requests. All nodes store operating intervals when they must be turned on for a certain duration. Figure 5 illustrates the process of adding an operation interval to a slave node. Slaves obtain the time instant in which the cost is minimized by asking the master node, which is responsible for applying the scheduling algorithm.

### 4.2. External Network

**Figure 6:**
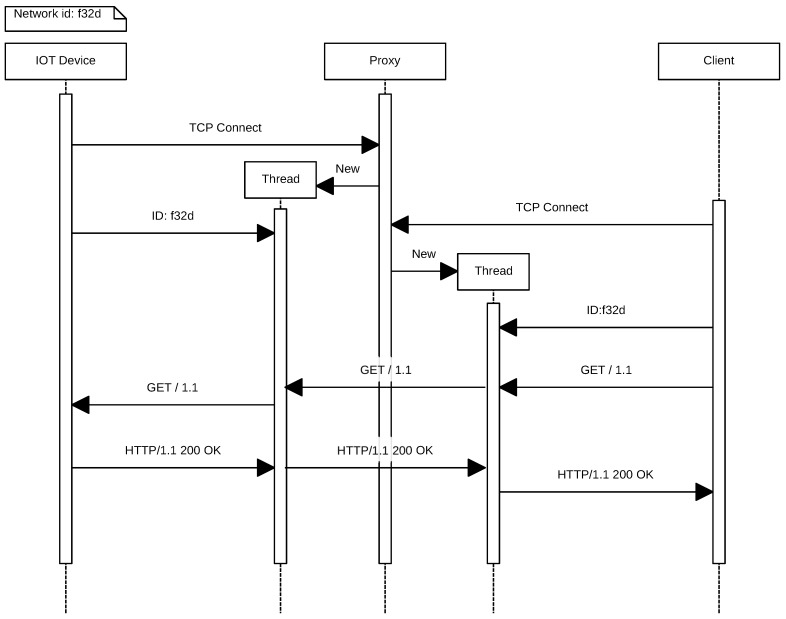
Sequence diagram that illustrates the tasks performed by the proxy server.

With respect to the external network, it is necessary to be able to send requests from the Internet into the IoT network, taking into account that it may be protected behind a firewall that prevents incoming connections or that the nodes may be in an NATnetwork. The solution proposed to solve this issue is illustrated in the sequence diagram shown in Figure 6. Such a solution consists of implementing a server that acts as a proxy and that stores persistent connections from the masters of each network. Thus, using an ad hoc protocol, the system can forward client requests to one of the open connections on the server and thus penetrate the target network. To achieve such a goal, a REST service converts HTTP requests so that they can be sent through the proxy, which translates them into the format of the ad hoc protocol previously mentioned.

It can be also observed in Figure 6 that the process begins when an IoT device opens a permanent connection to the proxy and authenticates indicating its ID (in this case, *f32d*). Then, the remote client opens a connection to the proxy server and authenticates with the same node ID (i.e., *f32d*). From this moment on, the remote client is able to send HTTP requests to the IoT device. Note that, instead of an HTTP request, any other type of protocol could be used. At this point, in the example described, the IoT node would receive the GET request, and it would send back the response.

### 4.3. REST Web Service

In order to integrate the system easily with data visualization applications, it was decided to implement an REST web service that is responsible for translating HTTP REST requests to the internal protocol to communicate them through the proxy. This service allows for integrating web/mobile/desktop applications without the need for implementing a specific protocol for each one of them.

In addition, this service is responsible for cleanup requests, acting as the first filter before the data enter the IoT network, thus removing headers that are not strictly necessary and that would increase the computational load of the IoT devices (i.e., they would increase energy consumption and would slow down the processing).

Since the REST service is completely separated from the proxy server, it is easy to maintain it. The service also gives the user the flexibility to connect third-party developments to the system, and it could be even used to interconnect nodes of networks located in different network segments.

**Figure 7:**
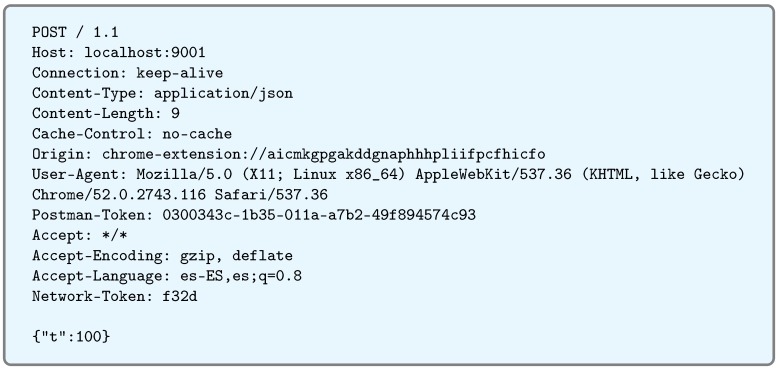
HTTP GET request.

**Figure 8:**
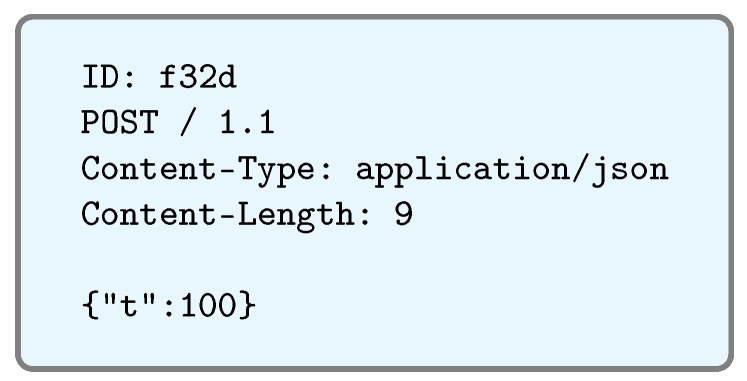
Optimized request.

**Figure 9:**
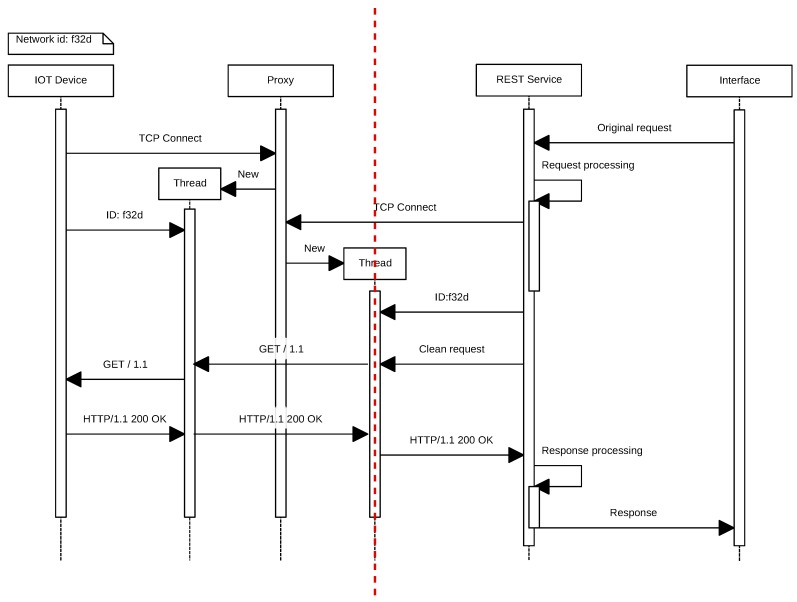
Sequence diagram that illustrates the tasks carried out by the REST service.

Figure 7 shows an example of the GET request processed by the service. Such a request was sent by a web client (in this case, PostMan [67]). As can be observed, the request is very verbose, and most of the information received is not useful for the IoT network. In fact, in this case, it is counterproductive, because it will force the IoT device to process a much larger amount of data, and because it is an integrated device with a limited computational capacity, it would lead to a considerable increase in response time. Therefore, the first action to be performed is to clean the request and forward only those data that will be relevant to the IoT device. The request, once filtered by the REST service, would look like the one shown in Figure 8. In Figure 9, the actual flow of the process previously described can be observed in the form of a sequence diagram.

## 5. Components of the System

**Figure 10:**
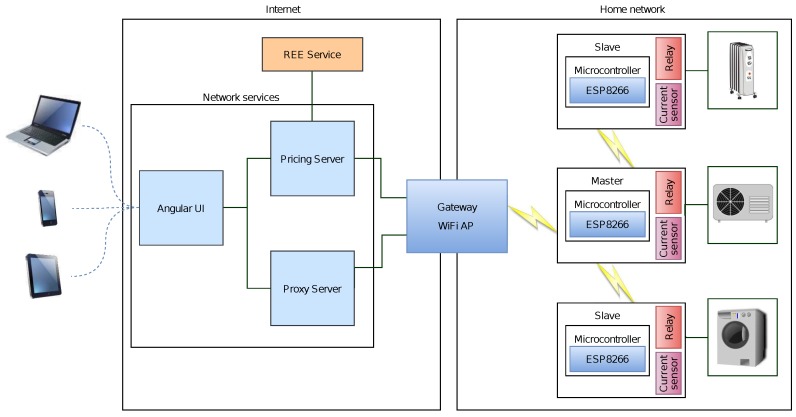
Components of the smart socket system.

As can be observed in Figure 10, each node of the IoT network consists basically of an ESP8266 module, a relay that allows for activating or deactivating the current flow, and an alternating current sensor that collects data of the power consumed by the smart power outlet. Furthermore, each node has a 230 V–5 V transformer able to use up to 700 mA, enough for powering up the ESP8266 (it may consume up to 240 mA), relay (72 mA) and the current sensor (10 mA).

The ESP8266 module acts as a control and communications subsystem. It is responsible for sending and receiving data from other nodes and for performing actions on sensors and actuators. In addition, one of the nodes in the network is automatically configured as the master, so it will perform control and planning tasks on the network.

As was previously illustrated in Figure 1, all of these components are grouped into different subsystems, which are detailed in the next subsections.

### 5.1. Sensor and Actuation Subsystem

#### 5.1.1. Current Sensor

This sensor detects the current flowing through a conductive element and generates a signal proportional to such a flow. The signal may consist of an analog voltage, an electric current or a digitally-encoded output. There are mainly four different types of current sensors: the ones based on Ohm’s law, sensors based on Faraday’s law, magnetic field-based sensors and Faraday-effect sensors [68]. A Hall-effect current sensor was chosen that met the specifications of the power outlet. Since the power outlet system was conceived of for small loads in home automation environments, the maximum power was established in 2000 W. Therefore, in a 230 V AC system (actually, in the Spanish regulations, it is 230 V ± 7%, so the voltage can reach almost 250 V), the limiting current will be approximately 8.127 A. Moreover, it is important to take into account that the control circuit that carries out the current measurements is operated at 5 V. Furthermore, for safety reasons, it is essential to insulate the 230 V-operated power circuit from the control circuit.

Among the different current sensors on the market, the Allegro ACS712-20AT was selected [69]. Such a sensor is powered with 5 V; it tolerates a maximum current of up to 30 A; it has a low energy consumption (10 mA) and a low cost (around $5, as of writing). In order to obtain with the sensor the effective value of the current flowing through a circuit, the mean squared value of the signal obtained at a time instant *t* has to be computed, which is proportional to the input current flow. For such a computation, the sensor signal has to be sampled during an interval *T*, as is shown in Equation (5), where $t_{0}$ is the time instant when sampling begins.

(5) $$U_{ef}=\sqrt{\frac{1}{T}\int_{t_{0}}^{t_{0}+T}i^{2}\left(t\right)dt}$$

#### 5.1.2. Relay

A relay is an electrical switch that allows for controlling, through a low-voltage circuit, a circuit of greater voltage, keeping them completely insulated electrically. Among all of the different types and models, a Songle SRD5V [70] was selected for the implementation described in this paper. It tolerates the maximum power established (2000 W); its control voltage is 5 V; and it has low-coil power consumption (360 mW) and a low-price (around $0.30 as of writing).

### 5.2. Control and Communications Subsystem

Sensors and actuators require an element to control them. Such an element has to be also responsible for collecting the data and for communicating with the other nodes of the system. For such tasks, a microcontroller gives a good trade-off between features and energy consumption.

Among all of the possible alternatives, it was decided to use the ESP8266 [71]. Specifically, during development a NodeMCU V1.0 board was used [72], while for the final assembly, a WeMos D1 Mini [73] was integrated in a power strip. Both boards use basically the same hardware and software, but the WeMos allows for reducing the form-factor of the prototype.

NodeMCU boards are really inexpensive (around $5, as of writing) and are as powerful as the other solutions analyzed. Since Wi-Fi is completely integrated on the board, no external shields or antennas are required. NoceMCU V1.0 contains a high-performance low-energy consumption module designed to be integrated in mobile developments. The module implements a full TCP/IP stack and offers wireless connectivity to other devices. Specifically, the module contains an ESP8266 System-on-Chip (SoC) from Espressif Systems [74] that has a 32-bit Tensilica LX106 Diamond Series processor at 80 MHz, with 50 KB of SRAM and 4 MB of flash memory. According to the manufacturer, 20% of the MIPS are used by the wireless protocol stack, while the rest is available for user applications.

ESP8266 modules are sold with a pre-installed firmware that receives AT commands through a serial port and that allows for performing basic configuration operations to use the module as a wireless connectivity interface for other devices. However, users can overwrite the flash memory with their own programs to perform more advanced operations.

Due to the low cost and the possibilities offered by this module, a large community of users has been created around it, and a multitude of free firmwares have been developed in order to extend the predetermined commands. Moreover, the community has developed environments based on the Arduino software to facilitate the programming of new firmware and to upload them into the flash memory of the device [75].

### 5.3. System Integration

**Figure 11:**
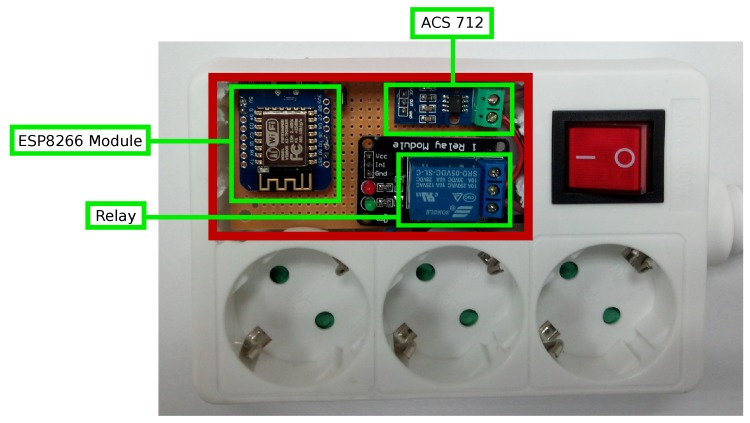
Final assembly and main components of the smart power outlet.

In the final assembly of the prototype, a modified five-way square power strip was used. Removing two of the power outlets leaves enough space for putting the assembly of all of the components of the module safely. Figure 11 shows the different components inside the power strip. As was mentioned previously, in order to reduce the size of the prototype, the NodeMCU board was replaced with a WeMos D1 Mini board, since both are almost identical in terms of features, but the WeMos is packaged in a more compact form-factor.

## 6. Implementation

### 6.1. Implemented Functionality

As was previously mentioned, the main functionality of the system proposed is based on its ability to obtain the hourly cost data from the REE Application Programming Interface (API) [76] in order to carry out a planning to switch on and off the power outlets at the time instants with less economic cost. In addition, the system is able to be configured remotely to change the working mode of each of the nodes; add, modify or delete periods of operation; and remotely power on and off on demand. For performing such tasks, the following functions were implemented:

- It allows for the configuration of each individual module in order to modify the network parameters or to add new nodes to an already existing network.
- Once a node is added to a specific network, such a network is able to self-reconfigure and self-manage automatically with the objective of providing network communications to the added node.
- It obtains automatically and on a daily basis the electricity price by using the REE API. The system also shows in real time the cost of the energy.
- The system is able to switch on and off every node of the IoT network in real time. It also shows the state of every power outlet in the network.
- The user can visualize remotely the historical energy consumption values of every IoT node.
- A user can change remotely the operation mode (i.e., manual or intelligent operation) of each power outlet. In intelligent mode, the system automatically switches on and off a node according to the time intervals that minimize the electricity cost.
- The user can define, show and modify the operation intervals for every power outlet.
- The system can be accessed from remote networks (i.e., from the Internet), being able to work behind a router that uses NAT without requiring additional configuration from the user.
- A REST API was implemented to offer access to the system to third parties, who can interact dynamically with it and develop new applications.

### 6.2. Initial Network Configuration

Setting up the system proposed is straightforward if there is an already existent Wi-Fi network. However, it is common to find routers that use NAT, which impedes the traffic from going directly through the router. As was detailed in Section 4.2, the solution to this problem consisted of implementing a TCP-level proxy server that accepts and stores persistent connections from the master nodes of each IoT network. Thus, by designing an ad hoc protocol above TCP, real-time requests can be sent to the master node through the already open connection. Then, this traffic can be redirected to the corresponding node according to the parameters defined by the protocol.

Regarding the initial configuration of each IoT node, it was decided that each node offers by default a WPA2-protected Wi-Fi network with a factory-defined random password. When a user connects to the network, each node offers access to the configuration API through an HTTP server. This approach is similar to the one offered by some commercial devices [50,52].

### 6.3. Obtaining the Electricity Price

**Figure 12:**
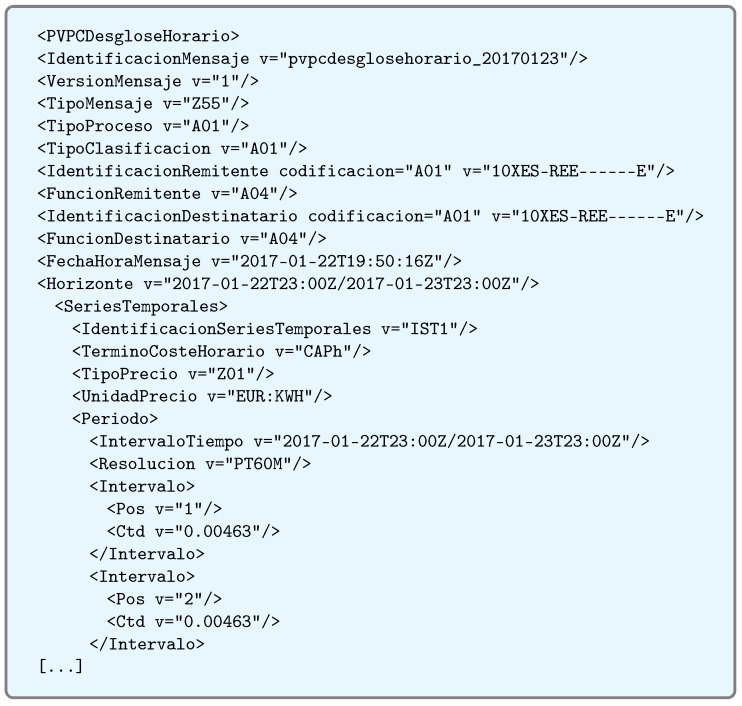
Original XML verbose format.

In order to obtain the electricity daily prices, making requests from the master node directly to the REE was initially considered API. However, after the first tests, this approach was ruled out because of its multiple problems:

- First, prices are supposedly published by law every day at the same time. However, in practice, the publication is delayed systematically. This fact derives having to make periodic requests until the resource becomes available. This is not a problem if a single node makes the requests, but in the case of multiple networks, each master would start polling at the same time, which is not the optimal solution in terms of energy consumption and communications traffic.
- Second, price lists are published in a very verbose XML and with too much information that is not relevant to the system proposed. An example can be seen in Figure 12. Such a large amount of information makes the data file require more than 40 KB, an amount that may seem to be acceptable at first glance, but for an integrated system with only 96 KB of memory (in which more than 50% is used by the network protocols implemented by the manufacturer), it becomes virtually impossible to handle.

**Figure 13:**
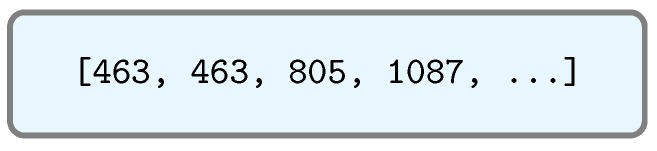
Pre-processed JSON format.

For such reasons, it was decided to implement the intermediate server detailed in Section 4.2, which cleans the XML data and converts them into a JSON file that is compressed and much less verbose. In this way, request polling is managed by the intermediate server, and IoT nodes receive a pre-processed and much lighter response, which allows them to evaluate and store data more quickly. Thanks to this approach, the resulting file is really small. For instance, Figure 13 contains the JSON file generated from the XML shown in Figure 12: the size of the processed JSON file is only 6.1% of the original XML file size.

It is also worth mentioning that this intermediate server allows for quickly adapting the system in case the REE API changes the syntax of the XML file. Thus, since all of the processing is carried out by the intermediate server, it is not necessary to update the firmware of the smart sockets, just the software of the server.

### 6.4. Visualization and Control Software

In order to implement the remote data visualization and control system, a web application was developed. Such a web page consisted of a single page application [77] developed using Angular2 rc5 [78]. Angular was selected due to the need for loading the data asynchronously, avoiding that the user unnecessarily waits to view the information.

**Figure 14:**
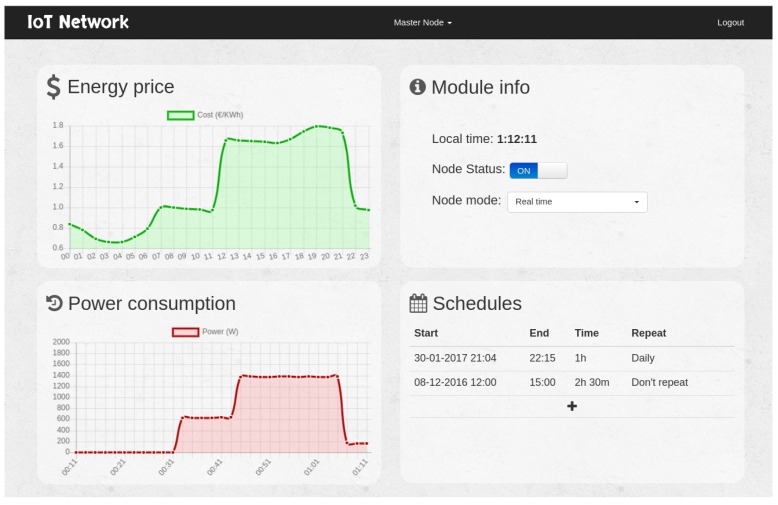
Main control panel.

The application was developed following the design recommendations for Angular2. Each module of the application was implemented in an Angular component that loads independently on demand. All components are asynchronous, and the application delegates in them all of the responsibility for obtaining the data and drawing the corresponding fragment of the interface autonomously. The interface has two modes (with and without logging in), which are managed by the presence of a session cookie. If the session has not been previously started, the login form is loaded. In such a form, the user must enter the token of the IoT network with which he or she wants to establish the communication. When the user enters the authentication token, a cookie is saved in the user’s browser, and the page is dynamically changed to show the components of the control panel (in Figure 14).

It should be noted that a task queue had to be established in the main control panel to limit the number of web components that can send requests simultaneously. It must not be forgotten that the system manages resource-constrained IoT devices, which would be saturated if the system sends too many requests in parallel.

**Figure 15:**
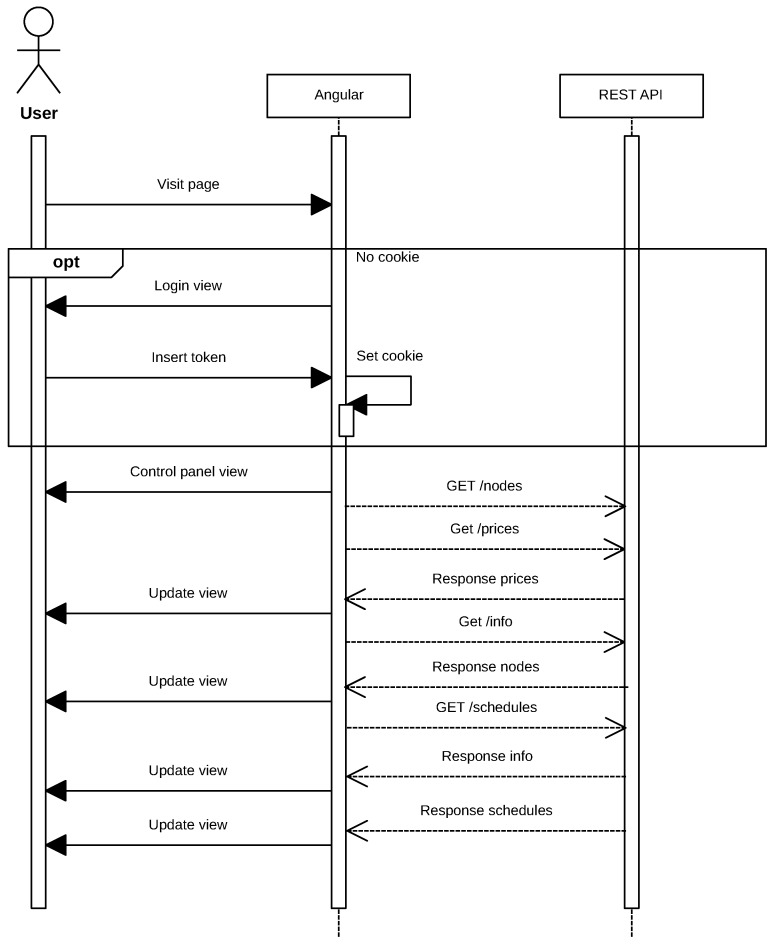
Web application sequence diagram.

In Figure 15, the flow of the application can be observed when a user visits the page. The requests exchanged between the Angular application and the REST API are performed asynchronously, but, as was previously indicated, requests are limited to avoid saturating the IoT devices. It must be noted that, although in the diagram shown in Figure 15 it is not specified for the sake of clarity, the REST API makes requests to the smart plugs linked to the token introduced by the user through its own communications protocol, as was previously described in Section 4.3.

## 7. Experiments

### 7.1. Initial Software Setup

Before performing any experiment, it is necessary to deploy the IoT network infrastructure. For such a purpose, the first step consists of deploying all of the services developed for communicating with the different subsystems.

The smart socket system presented in this paper is open-source, and all of the code can be found in [79]. This public repository can be cloned in order to deploy all services locally. After downloading the source files, the deployment is really straightforward and only requires two steps: first, a Docker [80] image has to be built by using the command *docker build*, and second, the Docker container has to be started with the command *docker run*.

### 7.2. Initial Configuration of the Nodes

Every new node to be added to system has to be configured to connect to a Wi-Fi access point. If the node detects that there are no other nodes in the network, it takes the role of master, connects to the access point and opens a persistent connection to the proxy server.

**Figure 16:**
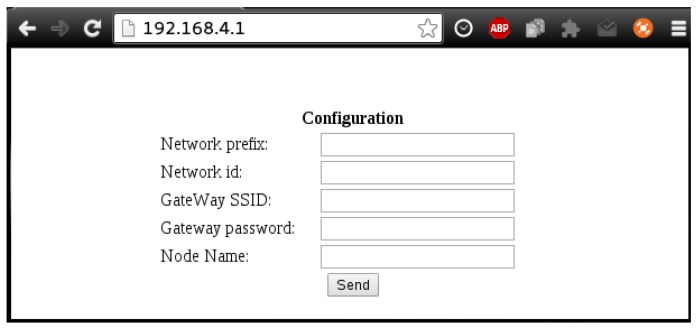
Initial configuration form of a smart socket.

When the node’s power supply is first connected, it creates a Wi-Fi network with a random password that would be assigned by the manufacturer. Then, a user can connect to the Wi-Fi network created by the IoT master node and access the default IP of the embedded HTTP server (192.168.4.1). The server shows a form where the user has to indicate the network configuration data (in Figure 16), which include the name of the Wi-Fi network to be connected to (i.e., the network with Internet access) and its password. Moreover, several default parameters can be customized, like the node’s name, the network id and the network prefix. In the case of the network id, it is used as a simple authentication token within the control panel, while the network prefix determines whether two nodes belong to the same network.

### 7.3. Accessing the Nodes through the REST API

**Figure 17:**
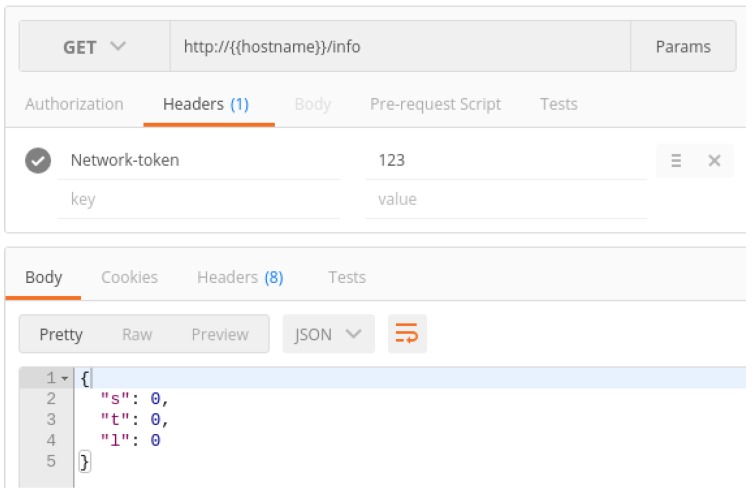
IoT network state request sent through PostMan.

Figure 17 shows an example of the REST API request that asks for information about the state of the network through PostMan. In such a request, the token of the IoT network whose data are being requested must be included (in the example shown in Figure 17 the token is “123”). The answer to the request is *{“s”:0, “t”:0, “l”:0}*:

- *“s”* stands for the state of the module, and it can be either 0 (off) or 1 (on).
- *“t”* represents the working mode. If it is equal to 0, the socket is working in intelligent mode, while 1 is for real-time (i.e., manual) mode.
- *“l”* represents the time when the last scheduling cycle took place. In the previous example, it is equal to 0, which means that it has never been a previous scheduling cycle.

### 7.4. Testing the Scheduler

**Figure 18:**
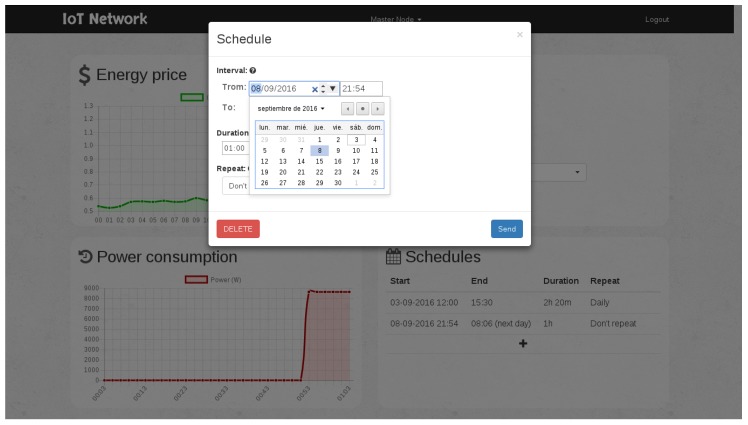
Example of scheduling for a one-hour operation interval.

**Figure 19:**
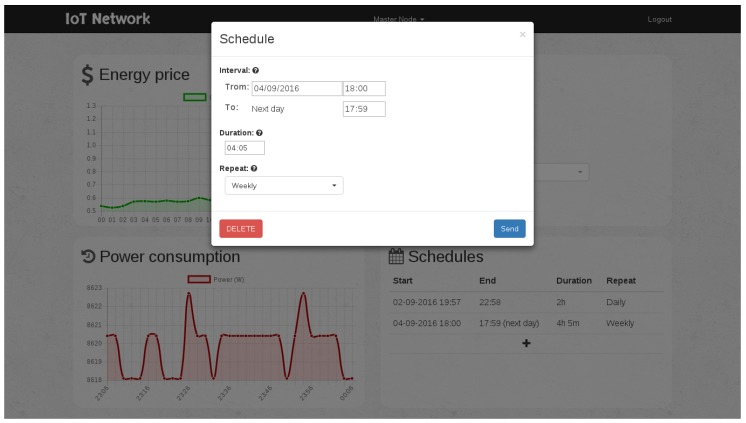
Menu for scheduling the operation intervals.

As an example, the scheduling module could be tested by adding a one-hour operation interval scheduled to begin within one minute. Figure 18 shows the menu where the scheduling is programmed. Thus, the scheduler is forced to switch on the power outlet after a minute, and then, it can be checked visually that it worked, for instance through a light connected to the power outlet. Note that, for this test, the scheduler had to be in manual mode in order to ignore the energy costs received. More complex schedules can be configured, as can be observed in Figure 19.

### 7.5. Communications Range

Indoor wireless communications are exposed to several problems because radio signals are reflected, refracted, absorbed and, therefore, attenuated by obstacles. In order to test the smart socket network infrastructure created in an indoor environment, it was setup, and several measures of the signal RSSI (Received Signal Strength Indicator) were taken from different locations during the regular operation of the modules. Moreover, five measurements were averaged for each spot to counteract signal fluctuations.

It was determined that the modules transmitted with a typical power of 18.5 dBm and that the intensity of the signal measured with line-of-sight at 3 m was −50 dBm. Such signal level decreased to −62 dBm when the socket was placed behind a brick wall. If the socket was located 6 m behind two brick walls, the intensity was reduced to −71 dBm. Therefore, taking into account that the ESP8266 usually requires a minimum of −80 dBm to guarantee communications, it can be concluded that, in spite of its small size, the range offered by the device is enough for a home automation environment.

### 7.6. Current Monitoring

In order to test the current monitor system in a real situation, the energy consumption of an electric heater with two power levels was measured. The first power level was rated at 700 W by the manufacturer, while the second one was at 1400 W.

**Figure 20:**
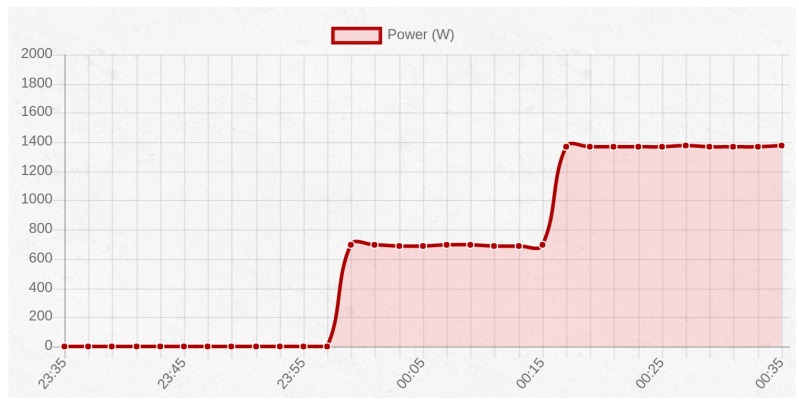
Graph representing the power consumption of an electric heater with two heat levels.

Figure 20 shows the real-time graph of the power consumption. First, the heater is switched on in the first level, and then, after fifteen minutes, it can be seen how the curve rises to 1400 W when the second heat level was selected. Therefore, it was concluded that the current intensity data collected are accurate both in value and timing.

### 7.7. Socket Performance with and without HTTPS

In order to analyze the performance and power consumption of the socket, several load tests were carried out for evaluating HTTP and HTTPS in real situations. All tests were performed with the device connected to a Wi-Fi in the IEEE 802.11g network mode with an output power of 15 dBm. The manufacturer rates the typical power consumption for this type of connection at 462 mW.

The tests performed applied different transaction load levels to a smart socket. Thus, the device first received a low load, which consisted of two requests per second. An average power of 463.65 mW was measured, both for HTTP and HTTPS, which is really close to the data indicated by the manufacturer.

**Figure 21:**
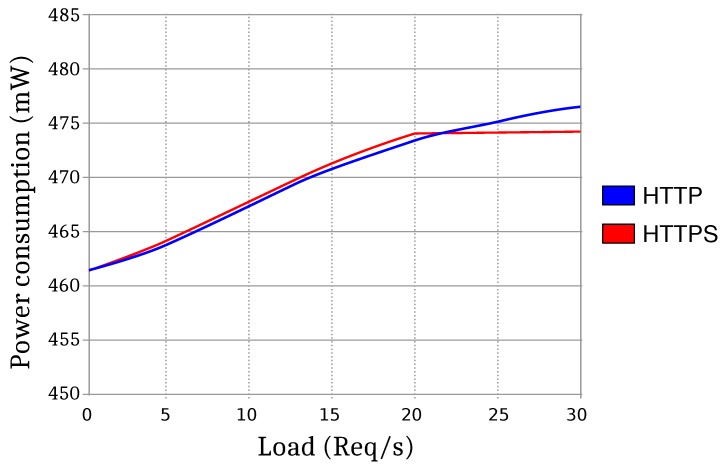
Graph comparing the power consumption of the device using HTTP and HTTPS.

In the second test, the load was increased to 21 requests per second. The average power rose to 474.87 mW. This was the maximum power that could be measured using HTTPS, because increasing the load further causes timeouts due to the overload of the device. However, when using HTTP, since less computation is required, the load could be increased to a maximum of 61.3 requests per second, and a power of 479.82 mW was reached. As a summary, Figure 21 shows the evolution of the power consumption when increasing the number of HTTP/HTTPS requests per second.

Regarding the performance in terms of processing delay, the differences between HTTP and HTTPS protocols were pronounced: in every test, HTTPS processing time was almost three-times slower than for plain HTTP due to the overhead of the key exchange and the computational cost associated with encryption.

### 7.8. Estimated Savings

This section shows an example that illustrates the cost savings that can be obtained thanks to the system proposed. It must be noted that, for the sake of clarity and simplicity, this example does not take into account time restrictions or a preferred order on the operation of the appliances (e.g., a dryer would be operated after the washing machine). Note also that the comparison excludes lighting and appliances like the oven, ceramic cook top or a TV because they are used on demand and cannot be deferred without disturbing the user preferences. Moreover, home appliances like refrigerators or freezers are not evaluated, since they have to be connected all day long. In addition, it has to be emphasized that the appliances selected are just examples, and their scheduling and use depend highly on the user preferences, his/her personal schedule and the place where the user lives (i.e., it may not be polite to switch on a noisy machine in the early morning in certain buildings).

Thus, Table 2 shows the potential savings that could be obtained by four different appliances when they are operated during the optimal cost intervals selected by the smart socket system. Specifically, the costs indicated refer to 31 January 2017 when using the default PVPC tariff. In such a scenario, the minimum *FEU* values were 0.12398 €/kWh (from 4:00–5:00) and 0.12596 €/kWh (from 3:00–4:00), whilst the maximum values were 0.16383 €/kWh (from 19:00–20:00) and 0.15999 €/kWh (from 18:00–19:00). These numbers indicate that a customer that operates a device at 4 a.m. saves 24.33% of the cost respect to one that switches on the same device at 7 p.m. Savings are more compelling when it is assumed that the energy consumption indicated is performed on a daily basis during a year: for the most energy-demanding appliances, savings of up to €67.41 can be achieved. The overall savings for these four appliances would reach €108.14 for a year of use.

### 7.9. Comparison to Other Energy Management Systems

The cost savings obtained in the previous section are in the same order of magnitude as the most relevant solutions found in the literature, which are compared in Table 3. It can be observed that some systems are developed specifically for optimizing the use of a specific appliance (or a set of appliances), but others, like the system proposed in this article, are aimed at providing a whole energy efficiency solution for smart homes. The features of the systems compared differ greatly, but most of them share similar objectives with the solution proposed: to increase energy efficiency and to incorporate user preferences. However, we have found no system that includes all of the functionality described in Section 6.1.

Regarding the architecture of the systems compared, most of them are HEMS that may include or not a WSN that collects data from different sensors, as does the system presented in this paper. The way energy prices are obtained is the main difference between the system proposed and the others analyzed: our system always make use of real prices obtained from a public operator, while other solutions try to predict price values or they just limit the consumption to off-peak consumption periods. Finally, in terms of cost savings, it is difficult to carry out a fair comparison, since they differ in many of the aspects previously mentioned: the technologies selected, the scheduling techniques, the systems modeled, the way energy prices are calculated and even in the possibility of using multiple energy sources.

## 8. Conclusions

The Internet of Energy (IoE) represents a novel paradigm of intelligent infrastructures where electrical power systems would work cooperatively with smart devices. The implementation of IoE systems involves the use of multiple software and hardware components like embedded systems, power electronics, integrated circuits, sensors, processing units or storage technologies. Such components are an essential part of the infrastructure of companies dedicated to the generation and distribution of energy and the one required by the final consumer. This article focused on the latter and presented a home automation system able to process the information about pricing and the user preferences to optimize home energy consumption. The system collects daily the data on pricing published by Red Eléctrica de España (Spanish Electrical Grid) and analyzes them to determine the operation time intervals with the lowest prices. To this end, an auto-configurable easy-to-deploy system based on a series of intelligent power outlets connected to each other through a shared Wi-Fi network was developed. Such an open-source smart socket system is able to measure the current of the appliances connected to it and send the data collected to a central control device that generates statistics and performs certain scheduled actions.

This article made three main contributions aimed at fostering IoT technologies for the IoE. First, it presents a hardware prototype and a software development able to test scheduling strategies by using actual price values from a public electricity operator. Second, the paper presented a thorough description of the design of the open-source system proposed, including its communications architecture, the protocols implemented, the main sensing and actuation components of the system and the most relevant pieces of the software developed. Third, the system proposed the features of self-organized mesh networking and auto-configuration characteristics that have not been found in the literature for any Wi-Fi smart plug.

To evaluate the performance of the smart socket system, different experiments were performed. The initial setup and configuration tests demonstrated that the open-source system is really easy to configure and deploy. The access for third-party developers was also tested, and it was shown that it is straightforward to use the REST API. Other experiments confirmed that the smart socket scheduling system and current monitoring features work fine and that the communications range is enough for home automation. The performance of the system when using plain HTTP and HTTPS was also compared: the results showed that, although in terms of average energy consumption there are no significant differences, the processing time required by HTTPS is roughly three-times greater than the one needed by HTTP, which denotes the need for more efficient and lighter ciphering suites for IoT devices. Moreover, an example was described that illustrates the cost savings achievable with the system proposed. In the scenario proposed for the experiments, an energy-demanding appliance can save up to €67.41 per year. Finally, the features of the system were compared with the most relevant energy management systems, concluding that the system offered similar energy savings, but more features than most of the systems analyzed.

## Figures and Tables

**Table 1:** Commercial smart plug comparison.

Model	Manufacturer	Communications Technology	Remote Control	Scheduling	Power Meter	Real-Time Pricing	Other Functionality	Price
MeterPlug	StickNFind	Bluetooth 4.0	Within 30 m	No	Yes	No	Stand-by detection Bluetooth proximity detection	In Pre-Order
DSP-W215	DLink	Wi-Fi	Yes	Yes	Yes	No	Overheat protection	$ 39.99
MyModlet	ThinkEco	Wi-Fi	Yes	Yes	Yes	No	-	$ 50 (Starter Kit)
MyPlug 2	Orange	GSM/GPRS	Yes	No	Yes	No	Blackout detection	€79
Energy Safety Outlet	SafePlug	ZigBee	No	No	Yes	No	Electrical shock and fire prevention, stand-by detection	$ 66
Neo	Ankuoo	Wi-Fi	Yes	No	No	No	Amazon Echo support	$ 19.99
SP-1101W	Edimax	Wi-Fi	Yes	Yes	No	No	-	€40
SWO-SMP1PA	SwannOne	ZigBee, Wi-Fi	Yes	Yes	Yes	No	-	$ 64.99
Circle	PlugWise	ZigBee	Yes	Yes	Yes	No	-	€360 (Home Basic Kit)
WeMo	Belkin	Wi-Fi	Yes	Yes	No	No	-	€49.99
S20	Orvibo	Wi-Fi, RF 433 MHz	Yes	Yes	No	No	-	$ 19.99

**Table 2:** Savings obtained for different appliances.

Appliance	Average Power (KW)	Duration (h)	Max. Cost (€/day)	Min. Cost (€/day)	Annual Savings (€)
Dishwasher	1.3	1	0.212979	0.161174	18.9088
Washing machine	0.5	1	0.081915	0.06199	7.2726
Dryer	2	0.5	0.16383	0.12398	14.5452
Air conditioning/heating	2.5	2	0.80955	0.62485	67.4155

**Table 3:** Main characteristics of the most relevant energy management systems.

System	Scenario	Objective	Architecture	Price source	Cost savings
Shajahan et al. [19]	Plug	Monitoring remote devices	Not defined	Price unawareness	When unwanted loads are turned off, authors claim an energy saving of 15%
Choi et al. [28]	Smart office	Control the power state of a user’s PC and the switching of the lights	Location-aware approach based on BLE beacons, smart plugs and a mobile app	Price unawareness	Experimental results over a three-month period showed average energy savings of 31.9% for the PCs and 15.3% for the lights
Rastegar et al. [39]	Washing machine, dryer and dishwasher	Incorporate priorities of on/off controllable appliances	HEMS	Three-level Time-of-Use (TOU) tariffs and Inclining Block Rate (IBR) pricing	Savings of 7.5% while respecting user preferences
Siebert et al. [40]	Two offices, multimedia set and washing machine	Compare centralized and decentralized smart plug networks	Different approaches to smart plug scheduling in HEMS	Brazilian opt-in TOU tariff for weekdays with three levels: off-peak, intermediate-peak and peak	The system is able to detect devices in stand-by mode, saving almost 13 h of stand-by consumption per day and device
Tsai et al. [41]	Home	DC power monitoring system and sensor network	ZigBee-based smart socket network	Re-scheduling between peak and off-peak hours	Savings between 10%–15% are achieved for a single demo room with proper setting and scheduling
Shie et al. [42]	Home	Smart energy monitoring system	ZigBee-based smart socket network	Price unawareness	Not specified
Babu et al. [43]	Intelligent home controller	Remote interface controller that includes customer preferences for loads. Its decisions are based on dynamic tariff rates using fuzzy logic	HEMS	Three-level TOU tariffs: off-peak, intermediate-peak and peak	Not specified
Karfopoulos et al. [44]	Home	Distributed demand management mechanism with QoE	HEMS with a coordination mechanism that evaluates in real-time the stochastic behavior of the customer and the intermittency of the distributed RES production	Not specified	Not specified
Pilloni et al. [45]	Washing machine, dishwasher, dryer, electric oven, HVAC and water heater	Optimize user profile preferences and consumption in off-peak periods	QoE-aware SHEM (Smart HEM) system	Scheduling appliances using TOU electricity prices of ENEL or maximizing renewable energy use	QoE case achieves up to 22% without Renewable Energy Sources (RES) and 30% with RES. QoE-unaware case, 33% without RES and 46% with RES
Du et al. [46]	Water heater	Create an appliance commitment algorithm that schedules thermostatically controlled household loads based on price and consumption forecasts considering QoE	Two-step linear sequential multi-loop algorithm	Forecasted, one day-ahead from the market clearing price	Savings of up to 21% when respecting users preferences, and up to 75% without respecting them
Jo et al. [47]	HVAC	Schedule considering QoE as well as the characteristics of thermal appliances	HVAC scheduling method for HEMS	Hybrid-power: electricity price and natural gas price based on the TOU and natural gas rates	Savings of up to 36% when setting a temperature range between 19 °C in 21 °C
Chen et al. [49]	Smart home	Minimize the electricity cost while satisfying user preferences	CPS (GPS, sensors, bio-sensors)	Real-time pricing published by PJMin the United States	Electricity cost can be reduced 14% on average

## Abbreviations

The following abbreviations are used in this manuscript:

API	Application Programming Interface
BLE	Bluetooth Low Energy
CPS	Cyber-Physical Systems
DR	Demand Response
Ep	Energía consumida en el período tarifario p
FPU	Término de Facturación de Potencia Unitaria
HEMS	Home Energy Management System
IoE	Internet of Energy
IoT	Internet of Things
MCF	Margen de Comercialización Fijo
NTP	Network Time Protocol
PLC	Power Line Communication
PVPC	Volunteer Price for the Small Consumer
REE	Red Eléctrica de España
REST	Representational State Transfer
RF	Radio Frequency
RFID	Radio Frequency IDentification
RTC	Real Time Clock
SoC	System-on-Chip
TPU	Término de Potencia Unitario
UPS	Uninterruptible Power System
WSN	Wireless Sensor Network
